# Long non-coding RNA KCND1 protects hearts from hypertrophy by targeting YBX1

**DOI:** 10.1038/s41419-023-05852-7

**Published:** 2023-05-30

**Authors:** Rui Yang, Liangliang Li, Yumeng Hou, Yingnan Li, Jing Zhang, Na Yang, Yuhan Zhang, Weihang Ji, Tong Yu, Lifang Lv, Haihai Liang, Xuelian Li, Tianyu Li, Hongli Shan

**Affiliations:** 1 Shanghai Frontiers Science Research Center for Druggability of Cardiovascular noncoding RNA, Institute for Frontier Medical Technology, Shanghai University of Engineering Science, Shanghai, 201620 China; 2 Department of Pharmacology (State-Province Key Laboratories of Biomedicine-Pharmaceutics of China, Key Laboratory of Cardiovascular Research, Ministry of Education), College of Pharmacy, Harbin Medical University, Harbin, 150081 China; 3 Department of Pharmacology, School of Basic Medicine, Inner Mongolia Medical University, Hohhot, 010110 China; 4 Center for Tumor and Immunology, the Precision Medical Institute, Xi’an Jiaotong University, Xi’an, 710115 China; 5 Department of Basic Medicine, The Centre of Functional Experiment Teaching, Harbin Medical University, Harbin, 150081 China; 6 Research Unit of Noninfectious Chronic Diseases in Frigid Zone (2019RU070), Chinese Academy of Medical Sciences, Harbin, 150081 China

**Keywords:** Mechanisms of disease, Cardiovascular diseases

## Abstract

Cardiac hypertrophy is a common structural remodeling in many cardiovascular diseases. Recently, long non-coding RNAs (LncRNAs) were found to be involved in the physiological and pathological processes of cardiac hypertrophy. In this study, we found that LncRNA KCND1 (LncKCND1) was downregulated in both transverse aortic constriction (TAC)-induced hypertrophic mouse hearts and Angiotensin II (Ang II)-induced neonatal mouse cardiomyocytes. Further analyses showed that the knockdown of LncKCND1 impaired cardiac mitochondrial function and led to hypertrophic changes in cardiomyocytes. In contrast, overexpression of LncKCND1 inhibited Ang II-induced cardiomyocyte hypertrophic changes. Importantly, enhanced expression of LncKCND1 protected the heart from TAC-induced pathological cardiac hypertrophy and improved heart function in TAC mice. Subsequent analyses involving mass spectrometry and RNA immunoprecipitation assays showed that LncKCND1 directly binds to YBX1. Furthermore, overexpression of LncKCND1 upregulated the expression level of YBX1, while silencing LncKCND1 had the opposite effect. Furthermore, YBX1 was downregulated during cardiac hypertrophy, whereas overexpression of YBX1 inhibited Ang II-induced cardiomyocyte hypertrophy. Moreover, silencing YBX1 reversed the effect of LncKCND1 on cardiomyocyte mitochondrial function and its protective role in cardiac hypertrophy, suggesting that YBX1 is a downstream target of LncKCND1 in regulating cardiac hypertrophy. In conclusion, our study provides mechanistic insights into the functioning of LncKCND1 and supports LncKCND1 as a potential therapeutic target for pathological cardiac hypertrophy.

## Introduction

Cardiac hypertrophy, characterized by cardiomyocyte enlargement, is a compensatory response to overload induced by physiological and pathological stimuli that cause cardiac contractile dysfunction and eventually leads to heart failure [1]. It is a common pathological change associated with many cardiovascular diseases, including arrhythmia [2]. Thus, appropriate interventions that may interfere with cardiac hypertrophy in the early stages are of great importance. Extensive research has implicated mitochondrial dysfunction in pathological cardiac hypertrophy [3, 4]. Mitochondria are involved in metabolic processes that are vital for cellular energy provision. Cardiac mitochondria generate ~95% of the adenosine triphosphate (ATP) used by the heart, indicating that cardiac mitochondria are key organelles that are crucial for cardiomyocyte energy transduction [5, 6]. These findings suggest that dysregulation of cardiac mitochondria may contribute to pathological cardiac hypertrophy and that regulation of cardiac mitochondrial function is a potential therapeutic target against cardiac hypertrophy.

Long non-coding RNAs (LncRNAs) are composed of more than 200 nucleotides and constitute mostly non-coding RNAs that do not encode proteins [7]. It mediates various physiological and pathological processes by recruiting transcription factors that act as microRNA sponges, regulate the translation of various mRNAs, silence transcription, and reprogram epigenetic processes [8, 9]. Recent studies have reported that LncRNAs play important roles in cardiovascular disease and are reliable diagnostic indicators as well as therapeutic targets for various cardiovascular diseases, including cardiac hypertrophy [10–12]. Accumulating evidence suggests that LncRNAs are dysregulated in cardiac hypertrophy and contribute to sarcomere construction, calcium channeling, and mitochondrial homeostasis in hypertrophic cardiomyopathy [1, 13, 14]. However, the role of LncRNAs in the treatment and diagnosis of cardiac hypertrophy remains unclear and requires further investigation.

In this study, we first identified a novel LncKCND1 that was found to be downregulated in pathological cardiac hypertrophy. Further analyses confirmed that overexpression of LncKCND1 protected mice from transverse aortic constriction (TAC)-induced cardiac hypertrophy and prevented Ang II-induced cardiomyocyte hypertrophy. Finally, we determined the molecular mechanisms underlying LncKCND1 function. By combining bioinformatics analysis and molecular experiments, we established that LncKCND1 regulates cardiac hypertrophy by directly binding to Y-box binding protein 1 (YBX1).

## Methods and materials

### Mice and animal models

C57BL/6 male mice (18–23 g) were purchased from Changsheng Biotechnology (Liaoning, China) and maintained in cages under standard conditions according to the regulations of the Institutional Animal Care and Use Committee of Harbin Medical University. LncKCND1 overexpression mice were generated by tail vein injection of adeno-associated virus serotype 9 (AAV9) carrying a vector or LncKCND1 (Viral Therapy Technologies, Wuhan, China) 4 weeks before surgery. The cardiac pressure overload model was established by TAC surgery for 4 weeks. As described previously [15], the mice were hooked up to a ventilator, and the thymus was cut open to expose the aortic arch. The 7-0 ligation line is threaded through the aortic arch, over which a 26-G dummy needle is placed, and the vessel is ligated. The mice were sacrificed, and the whole mouse heart was removed at 4 weeks after TAC.

### Echocardiography

The mice were anesthetized with sodium pentobarbitone (40 mg/kg, i.p.) and xylazine (12.5 mg/kg, i.p.) and placed on a platform. Four weeks after TAC, two-dimensional M-mode echocardiography was conducted using VINNO6 (VINNO Technology, Suzhou, China) before sacrifice. Left ventricular fractional shortening (FS) and ejection fraction (EF) were recorded.

### Histological analysis

Hearts were harvested, fixed with 4% paraformaldehyde for 48 h, and dehydrated before being embedded in paraffin. Tissues were then sectioned into 5 µm slides for hematoxylin and eosin (H&E) staining, Masson staining, and wheat germ agglutinin (WGA) (L4895; Sigma, St. Louis, MO, USA) staining, as described in a previous study [15]. H&E staining was performed according to the manufacturer’s protocol using an H&E stain kit (G1120, Solarbio, Beijing, China). Masson’s trichrome staining was performed according to the manufacturer’s protocol using Masson’s trichrome stain kit (G1340, Solarbio, Beijing, China).

### Cell culture and transfection

Mouse cardiomyocytes (CMs) were obtained from 1–3-day-old neonatal mice hearts. Briefly, mouse hearts were extracted and digested with pancreatin (C0202; Beyotime, Shanghai, China). The cells were then maintained in DMEM supplemented with 10% fetal bovine serum (Biological Industries (BI), Kibbutz Beit-Haemek, Israel), 100 U/mL penicillin, 100 μg/mL streptomycin, and 1 mmol/L BrdU. Cardiomyocytes were incubated at 37 °C in 5% CO_2_ for 48 h. CMs were transfected with plasmid or siRNA (Sigma, St. Louis, MO, USA) using Lipofectamine 2000 (Invitrogen, Carlsbad, CA, USA), and after 6 h, the medium was replaced with DMEM and an additional 1 μmol/L angiotensin II (A9847, Ang II, Sigma, St. Louis, MO, USA) was added for 48 h.

### Detection of reactive oxygen species (ROS) production

An ROS assay kit (CA1410; Solarbio, Beijing, China) was used to detect ROS production in CMs. Dichloro-dihydro-fluorescein diacetate (DCFH-DA) was used as a fluorescence probe for ROS. DCFH-DA was diluted in DMEM to a final concentration of 10 μM. Then CMs were washed with PBS three times and incubated with DCFH-DA for 20 min at 37 °C. DCFH-DA was removed, and CMs were washed with PBS. Cells were imaged using confocal microscopy (Zeiss, Germany) and fluorescence intensity was measured using Image-Pro Plus 6.0.

### Detection of mitochondrial membrane potential

The mitochondrial membrane potential assay kit with JC-1 (C2006, Beyotime, Shanghai, China) was used to assess the mitochondrial membrane potential of the CMs, according to the manufacturer’s protocol. JC-1 accumulates in the mitochondrial matrix at high potentials, emitted red fluorescence, and in contrast, emitted green fluorescence at low potentials. The state of the membrane potential was expressed as the ratio of red to green fluorescence. Cells were imaged using confocal microscopy (Zeiss, Germany), and fluorescence intensity was measured using Image-Pro Plus 6.0.

### Mitosox

Cells were stained according to the protocol of the mitochondrial reactive oxygen species detection kit purchased from Beibokit (BB-46091, Shanghai, China). The cells were added with 250 μL of pre-warmed BBcellProbe OM08 and incubated in the dark for 30 min, after which the culture medium was replaced. Nuclei were incubated with Hoechst for 7 min at room temperature. Cells were observed under a fluorescence microscope and fluorescence intensity was measured using Image-Pro Plus 6.0.

### Detection of adenosine triphosphate (ATP) levels

Intracellular ATP was detected using an ATP assay kit (S0026, Beyotime, Shanghai, China). Lysis buffer was added to the cells and the supernatant was collected after centrifugation at 12,000 g for 5 min. Next, 100 μL of supernatant was added to 100 μL of ATP detection solution, and ATP levels were measured using a microplate reader (Molecular Devices, San Jose, CA, USA). ATP content was normalized to the ATP protein content based on a standard curve.

### Detection of cell surface area

CMs were fixed with cold methanol for 30 min, permeabilized with 0.1% Triton-X100 for 1 h, and blocked with goat serum for 40 min. Subsequently, cells were incubated with primary antibody (α-actinin, ab9465, Abcam, Cambridge, MA, USA) overnight at 4 °C. After washing with PBS three times, the cells were incubated with the secondary antibody for 1 h. Finally, nuclei were stained with DAPI (C1002, Beyotime, Shanghai, China). Immunofluorescence was analyzed using a confocal microscope (Zeiss, Germany). The cell surface areas were measured using Image-Pro Plus 6.0.

### Western blotting

Total protein was extracted and separated using 10% sodium dodecyl sulfate-polyacrylamide gel electrophoresis (SDS-PAGE) gels. The proteins were then transferred to nitrocellulose membranes and blocked with 5% nonfat milk for 1 h. Afterward, membranes were incubated at 4 °C with primary antibodies overnight. The next day, the membranes were washed 3 times with PBST, incubated with the secondary antibody for 1 h, and finally scanned with an Odyssey Imaging system (LI-COR, NE, USA). The primary antibodies used were anti-β-MHC (ab180779, Abcam, Cambridge, MA, USA), anti-YBX1 (20339-1-AP, Proteintech, Wuhan, China), and anti-GAPDH (10494-1-AP, Proteintech, Wuhan, China).

### Quantitative real-time PCR (qRT-PCR)

Total RNA was extracted from cardiac fibroblasts (CFs), CMs, and heart tissues using TRIzol, according to the standard protocol. Reverse transcription was performed after quantity measurement using the All-In-One 5X RT MasterMix (Abm, Vancouver, Canada). The mRNA levels were determined by quantitative PCR using a LightCycler machine (ABI, CA, USA) and BlasTaq™ 2X qPCR MasterMix (Abm, Vancouver, Canada). The sequences of the primers were synthesized by Sangon Biotech (Shanghai, China) and are listed in Supplementary Table S1.

### RNA immunoprecipitation (RIP) assay

The RIP assay was performed according to the manufacturer’s protocol using the Magna RIP RNA-Binding Protein Immunoprecipitation Kit (17–700, Millipore, Billerica, MA, USA). The heart tissues were washed with cold PBS and lysed with RIP buffer. Magnetic beads were then conjugated to human anti-YBX1 antibodies or normal mouse immunoglobulin G (IgG; 16402-1-AP; Proteintech, Wuhan, China) to capture the RNAs used for qRT-PCR analysis. The expression levels of LncKCND1 were measured using qRT-PCR.

### RNA pull-down assay

RNA pull-down analysis was conducted using a Pierce Magnetic RNA-Protein Pull-Down Kit (20164, Thermo Scientific, Waltham, MA, USA). Protein samples (2 mg/mL) were collected for further use, and the target RNA was labeled using the Pierce RNA 3′ Desthiobiotinylation Kit (20163, Thermo Scientific, Waltham, MA, USA). Target RNA and streptavidin magnetic beads were mixed according to the manufacturer’s protocol. The protein was then bound to RNA using protein-RNA binding buffer and the master mix of RNA-protein binding reaction was prepared, incubated for 30–60 min at 4 °C with agitation, washed, and eluted with RNA-binding protein complexes by heating at 95–100 °C for 10 min for further western blot analysis.

### Mass spectrometry (MS)

RNA-binding protein obtained by RNA pull-down assay was separated by 10% gel, and the gel was stained with staining buffer (Coomassie blue) and de-stained until the bands of interest appeared. The relevant bands were removed for further MS analysis. MS was performed by Novogene Co., Ltd. (Beijing, China).

### Statistics

Data are presented as mean ± SEM. Significances were calculated using a two-tailed *t*-test, one-way analysis of variances (ANOVA), or rank sum test with post-test Bonferroni-corrected *t*-test as a post hoc test. *P* < 0.05 was considered to be statistically significant. Data were analyzed using GraphPad Prism 7.0.

## Results

### Downregulation of LncKCND1 in cardiac hypertrophy

**Fig. 1:**
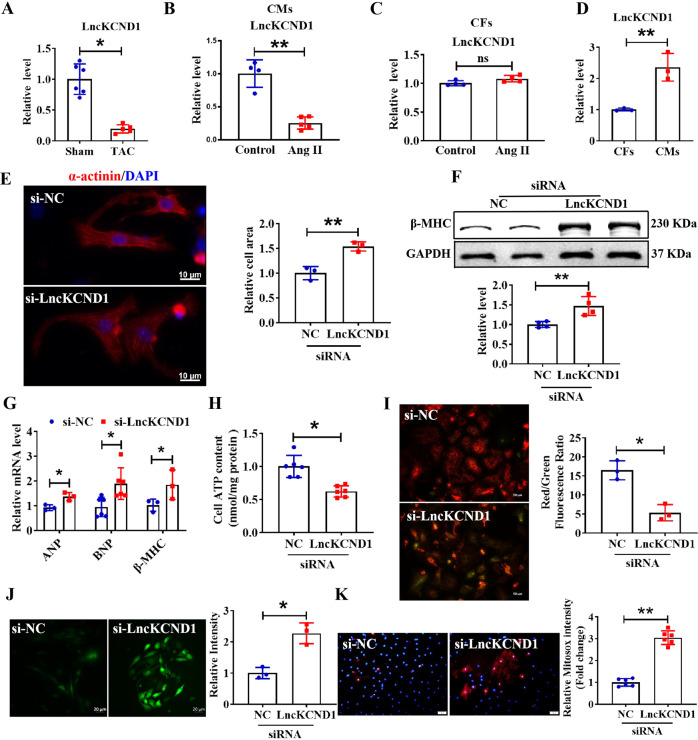
Inhibition of LncKCND1 promotes cardiomyocytes hypertrophy. **A** The relative level of LncKCND1 in hearts of Sham and TAC mice (*n* = 5, *P < 0.05 vs. Sham). **B** Downregulated expression level of LncKCND1 in Ang II-treated CMs (*n* = 5, **P < 0.01 vs. Control). **C** The relative level of LncKCND1 in Ang II-treated cardiac fibroblasts (CFs) (*n* = 4, **P < 0.01 vs. Control). **D** Relative level of LncKCND1 in the primary CFs and CMs (*n* = 3, **P < 0.01 vs. CFs). **E** Representative images of CMs transfected with si-NC or LncKCND1 siRNA for 48 h. Cells were stained by α-actinin and DAPI for nuclear staining (*n* = 3, **P < 0.01 vs. si-NC; scale bars, 10 μm). **F** Representative western blot band of the β-MHC level after silencing LncKCND1 (*n* = 4, **P < 0.01 vs. si-NC). **G** Relative mRNA levels of ANP, BN*P* and β-MHC in CMs transfected with LncKCND1 siRNA (*n* = 3 for ANP and β-MHC; *n* = 6 for BNP, *P < 0.05 vs. si-NC). **H** The effect of LncKCND1 silencing on CMs’ cellular ATP content (*n* = 6, *P < 0.05 vs. si-NC). **I** Representative images of CMs’ JC-1 staining after transfection with LncKCND1 siRNA for 48 h. The state of the membrane potential was expressed by the ratio of red to green fluorescence (*n* = 3, *P < 0.05 vs. si-NC; scale bars, 50 μm). **J** The ROS levels were detected in CMs’ transfected with LncKCND1 siRNA for 48 h. (*n* = 3, *P < 0.05 vs. si-NC; scale bars, 20 μm). **K** The mitochondrial ROS levels were detected in CMs’ transfected with LncKCND1 siRNA for 48 h (*n* = 6, **P < 0.01 vs. si-NC; scale bars, 50 μm).

First, we established a TAC model in C57BL/6 mice to determine the role of LncKCND1 in pathological cardiac hypertrophy. As shown in Supplementary Fig. S1A–C, compared to the sham-operated group, cardiac function in the TAC group decreased significantly with decreased left ventricular ejection fraction (LVEF) and fractional shortening (LVFS). The mRNA levels of atrial natriuretic peptide (ANP), brain natriuretic peptide (BNP), and β-myosin heavy chain (β-MHC) were upregulated in the hearts of TAC mice (Supplementary Fig. S1D). Furthermore, the protein expression level of β-MHC was significantly upregulated in the TAC mice (Supplementary Fig. S1E). We detected a series of differentially expressed LncRNAs in the hearts of mice with diabetic cardiomyopathy by gene chip analysis. Subsequently, these LncRNAs were further verified in the TAC model by qRT-PCR. Furthermore, qRT-PCR analysis showed that the expression level of LncKCND1 was decreased in hypertrophic heart tissues (Fig. 1A). In the in vitro assay, that involved primary cardiomyocytes (CMs) being treated with Ang II to induce hypertrophy. As shown in Supplementary Fig. S1F, cell size was significantly increased in the Ang II-treated group. Moreover, the mRNA and protein levels of hypertrophic markers were upregulated in Ang II-treated CMs (Supplementary Fig. S1G, H). Consistent with the in vivo results, the expression level of LncKCND1 was also decreased in Ang II-induced hypertrophic CMs (Fig. 1B). But the expression level of LncKCND1 remained unchanged in Ang II-induced hypertrophic CFs (Fig. 1C). Additionally, LncKCND1 showed higher expression levels in CMs than in cardiac fibroblasts (CFs) (Fig. 1D), indicating that LncKCND1 may play an important role in regulating pathological cardiac hypertrophy through CMs.

### Silencing of LncKCND1 promotes cardiomyocytes hypertrophy

To determine the role of LncKCND1 in cardiac hypertrophy, we transfected small interfering RNA (siRNA) into primary cardiomyocytes from neonatal mice. First, we evaluated the knockdown efficiency of the three siRNAs and used the most effective siRNA-3 in our subsequent experiments (Supplementary Fig. S2A). Immunofluorescence staining of α-actinin demonstrated that silencing of LncKCND1 significantly increased the size of the cardiomyocytes, as well as the protein expression levels of β-MHC and mRNA levels of ANP, BNP, and β-MHC (Fig. 1E–G).

Knowing that cardiac mitochondrial impairment can lead to cardiac hypertrophy [16]; thus, we determined the cardiac mitochondrial integrity and function after silencing LncKCND1 through JC-1 staining, and quantified ROS and ATP production. As shown in Fig. 1H, silencing of LncKCND1 significantly reduced ATP production. JC-1 staining showed that the knockdown of LncKCND1 caused a tremendous depolarization of the cardiac mitochondrial membrane (Fig. 1I). Silencing of LncKCND1 also increased ROS production (Fig. 1J). Additionally, the silencing of LncKCND1 significantly increased mitochondrial ROS production (Fig. 1K). These results suggest that the LncKCND1 knockdown impairs cardiac mitochondrial function and leads to hypertrophic remodeling of cardiomyocytes.

### Overexpression of LncKCND1 inhibits cardiac hypertrophy

**Fig. 2:**
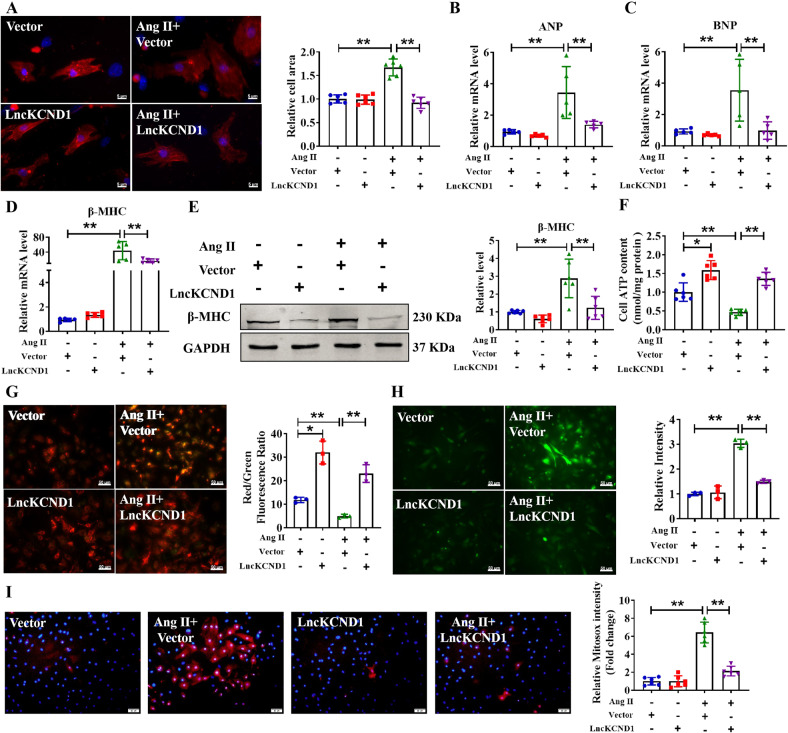
Overexpression of LncKCND1 inhibits Ang II-induced cardiomyocytes hypertrophy in vitro. **A** After CMs transfection with Vector or LncKCND1 for 6 h, Ang II was added for 48 h. Cells were stained by α-actinin and DAPI for nuclear staining (*n* = 6, **P < 0.01; scale bars, 5 μm). **B**–**D** Relative mRNA levels of ANP, BNP and β-MHC in CMs after transfection of LncKCND1 (*n* = 5, **P < 0.01). **E** Representative western blot band of the β-MHC after LncKCND1 overexpression under Ang II stimulation (*n* = 6, **P < 0.01). **F** Effects of overexpression of LncKCND1 on CMs’ cellular ATP content (*n* = 6, *P < 0.05, **P < 0.01). **G** Representative images of CMs’ JC-1 staining after LncKCND1 overexpression (*n* = 3, *P < 0.05, **P < 0.01; scale bars, 50 μm). **H** ROS level was determined in CMs transfected with LncKCND1 under Ang II stimulation (*n* = 3, **P < 0.01; scale bars, 50 μm). **I** Representative images of mitochondrial ROS staining after LncKCND1 overexpression (*n* = 6, **P < 0.01; scale bars, 50 μm).

To explore the anti-hypertrophic role of LncKCND1, we transfected LncKCND1 or an empty vector into CMs with or without Ang II treatment. As shown in Supplementary Fig. S2B, the expression level of LncKCND1 was significantly higher in the LncKCND1 transfection group than in the empty vector group. Administration of Ang II increased the cell surface area and promoted the mRNA levels of ANP, BNP, and β-MHC, and the protein expression level of β-MHC, whereas these effects were prevented by LncKCND1 (Fig. 2A–E). Also, overexpression of LncKCND1 alleviated mitochondrial dysfunction in Ang II-treated CMs (Fig. 2F–I).

**Fig. 3:**
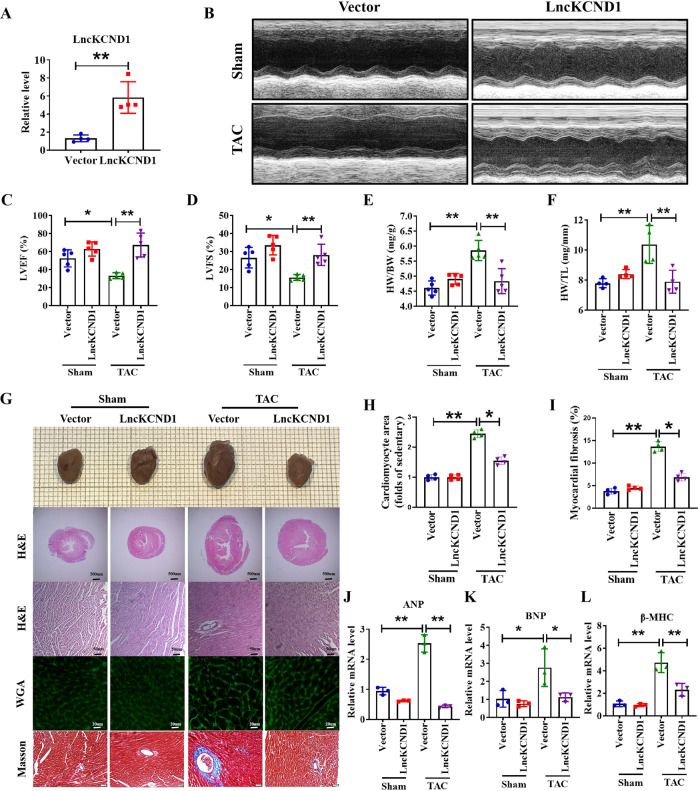
Overexpression of LncKCND1 mitigates pressure overload-induced cardiac hypertrophy in vivo. **A** LncKCND1 overexpression mice were generated by tail vein injection of adeno-associated virus serotype 9 (AAV9) carrying a vector or LncKCND1 for 4 weeks, and then subjected to Sham or TAC operation for 4 weeks (*n* = 4, **P < 0.01 vs. Vector). **B**–**D** Cardiac function of mice were determined by two-dimensional M-mode echocardiography (ECG). ECG assessment of left ventricle ejection fraction and fractional shortening of Sham or TAC mice infected with AAV9-Vector or AAV9-LncKCND1 (*n* = 5, *P < 0.05, **P < 0.01). **E**–**F** Comparisons of heart weight (HW)/body weight (BW) and HW/tibia length (TL) of Sham or TAC mice infected with AAV9-Vector or AAV9-LncKCND1 (*n* = 4-5, **P < 0.01). **G** Histological analysis of heart sections from Sham-Vector, Sham-LncKCND1, TAC-Vector and TAC-LncKCND1 groups. Five-µm-thick heart sections were stained with hematoxylin-eosin (H&E), wheat germ agglutinin (WGA) and Masson. **H**, **I** Statistics of cardiomyocyte size and myocardial fibrosis area (*n* = 4, *p < 0.05, **p < 0.01). **J**–**L** Relative mRNA levels of ANP, BNP and β-MHC in the heart tissues of Sham-Vector, Sham- LncKCND1, TAC-Vector and TAC- LncKCND1 groups (*n* = 3, *p < 0.05, **p < 0.01).

Next, to further examine the protective effect of LncKCND1 on pathological cardiac hypertrophy, mice tail vein injection of AAV9 viral particles carrying LncKCND1 or a vector was performed 4 weeks before the TAC operation. As shown in Fig. 3A, the expression level of LncKCND1 was markedly higher in the LncKCND1 group than in the vector group. Compared to the TAC group, forced expression of LncKCND1 improved cardiac function in mouse hearts at 4 weeks after TAC (Fig. 3B–D). Moreover, 4 weeks after TAC, hypertrophic responses such as heart weight (HW)/ body weight (BW) and HW/ tibia length (TL) were significantly upregulated, while overexpression of LncKCND1 partially prevented these effects (Fig. 3E, F). Histological analysis using H&E and WGA staining results showed a pronounced enlargement of the heart and CMs after TAC, which was attenuated after LncKCND1 overexpression. Additionally, the Masson staining assay showed that overexpression of LncKCND1 also decreased fibrosis levels (Fig. 3G–I). Similarly, the mRNA levels of ANP, BNP, and β-MHC in the LncKCND1-overexpressing mice also decreased after TAC compared to the vector group (Fig. 3J–L).

### LncKCND1 binds to YBX1 and promotes its expression in cardiac hypertrophy

**Fig. 4:**
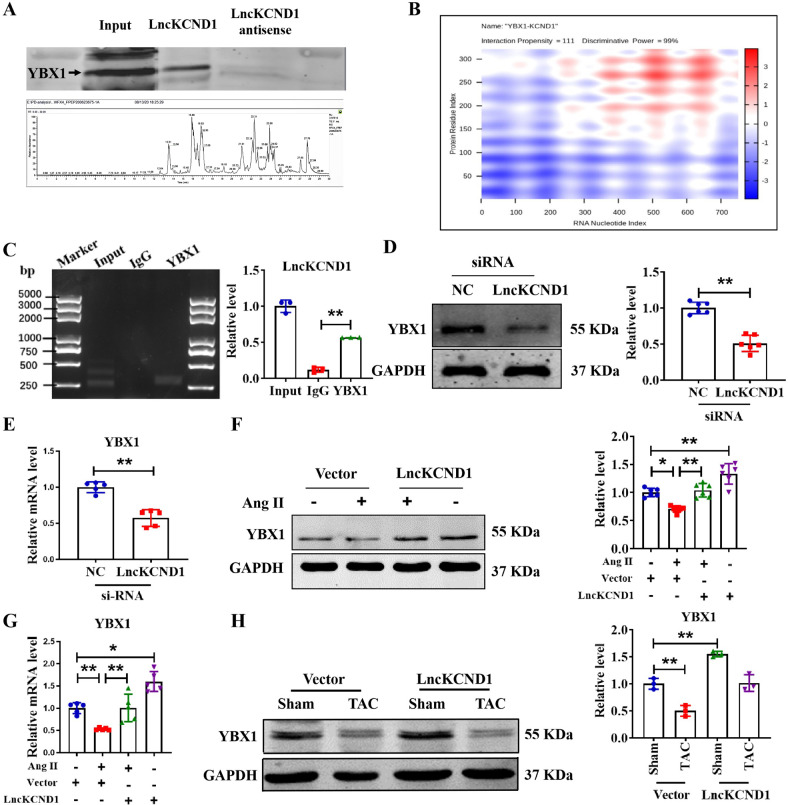
LncKCND1 binds to YBX1 and increases the expression of YBX1. **A** Representative western blot band of YBX1 protein pulled down by LncKCND1 (up). Lower: Extracted-ion chromatogram (XIC) for the YBX1 by MS (down). **B** Prediction of the overall interaction propensity between LncKCND1 and YBX1 by catRAPID. **C** RIP experiments were performed using YBX1 and negative IgG antibody. The purified RNA was used for further qRT-PCR assays (*n* = 3; **P < 0.01). **D** Representative western blot band of the YBX1 after transfection of LncKCND1 siRNA (*n* = 6, **P < 0.01 vs. si-NC). **E** Relative mRNA levels of the YBX1 after transfection of LncKCND1 siRNA (*n* = 5, **P < 0.01 vs. si-NC). **F** Representative western blot band of the YBX1 after overexpression of LncKCND1 under Ang II stimulation (*n* = 6, *P < 0.05, **P < 0.01). **G** Relative mRNA levels of the YBX1 after overexpression of LncKCND1 under Ang II stimulation (*n* = 5, *P < 0.05, **P < 0.01). **H** Representative western blot band of the YBX1 after overexpression of LncKCND1 under TAC surgery (*n* = 3, *P < 0.05).

To explore the mechanism by which LncKCND1 regulates cardiac hypertrophy, RNA pull-down assays and MS techniques were applied (Fig. 4A). Among all 156 proteins recognized by MS, we found that YBX1 could bind to LncKCND1. We also found predicted binding connections between YBX1 and LncKCND1 by searching the catRAPID database (http://service.tartaglialab.com/page/catrapid_group) (Fig. 4B). To further determine the relationship between LncKCND1 and YBX1, we next performed an RIP assay and found that LncKCND1 could be enriched in YBX1 compared to IgG, which showed the interaction between LncKCND1 and YBX1 (Fig. 4C). Furthermore, Silencing of LncKCND1 significantly inhibited the protein and mRNA expression level of YBX1 (Fig. 4D, E). In contrast, as shown in Fig. 4F, G, YBX1 was significantly downregulated after Ang II treatment when compared to the control group, while overexpression of LncKCND1 could increase the protein and mRNA expression level of YBX1. Additionally, YBX1 was significantly downregulated after TAC surgery, but this effect was significantly inhibited after LncKCND1 overexpression (Fig. 4H).

### Overexpression of YBX1 inhibits Ang II-induced cardiomyocyte hypertrophy

**Fig. 5:**
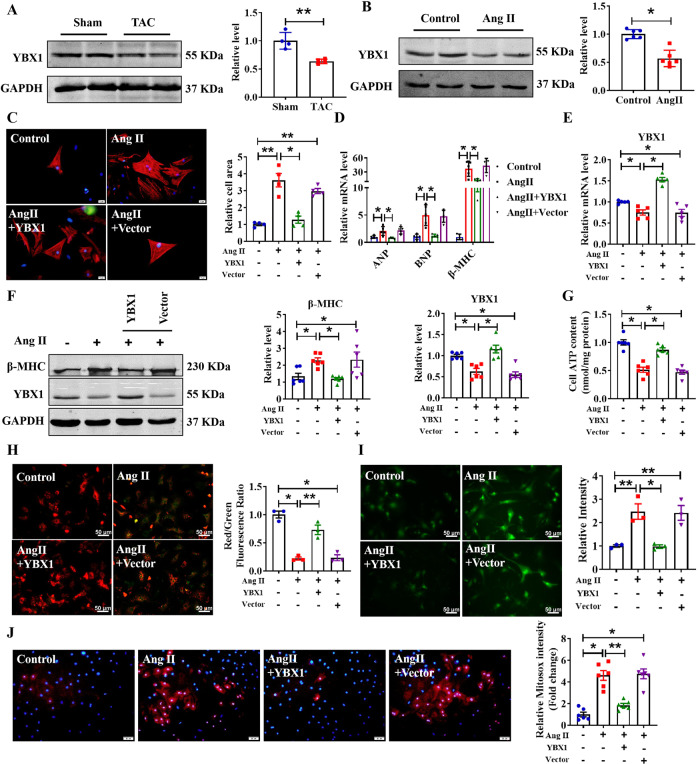
Overexpression of YBX1 attenuates cardiomyocytes hypertrophy. **A** The protein level of YBX1 in hearts of Sham and TAC mice (*n* = 4, **P < 0.01 vs. Sham). **B** Relative expression level YBX1 protein in primary cardiomyocytes induced by Ang II for 48 h (*n* = 6, *P < 0.05, vs. Control). **C** After CMs transfection with Vector or YBX1 for 6 h, Ang II was added for 48 h. CMs were stained by α-actinin and DAPI for nuclear staining (*n* = 4, *P < 0.05, **P < 0.01; scale bars, 20 μm). **D**, **E** Relative mRNA level of YBX1 and hypertrophy marker genes ANP, BNP and β-MHC in CMs after transfection of YBX1 (*n* = 5, *P < 0.05). **F** Representative western blot bands of the β-MHC and YBX1 after YBX1 transfection under Ang II stimulation (*n* = 6, *P < 0.05). **G** Effect of overexpression of YBX1 on CMs cellular ATP content under Ang II stimulation (*n* = 6, *P < 0.05). **H** Representative images of CMs’ JC-1 staining after YBX1 overexpression (*n* = 3, *P < 0.05, **P < 0.01; scale bars, 50 μm). **I** ROS level, determined in CMs transfected with YBX1, under Ang II stimulation (*n* = 3, *P < 0.05, **P < 0.01; scale bars, 50 μm). **J** Representative images of mitochondrial ROS staining after YBX1 overexpression under Ang II stimulation (*n* = 6, *P < 0.05, **P < 0.01; scale bars, 50 μm).

Although Huang et al. reported that YBX1 can promote the proliferation of CMs [17], its role in cardiac hypertrophy remains unclear. To clarify its regulatory role in pathological cardiac hypertrophy, we first measured the expression level of YBX1 in hypertrophic heart tissues and Ang II-induced CMs. As shown in Fig. 5A, B, YBX1 expression levels were significantly decreased under hypertrophic conditions. As shown in Supplementary Fig. S2C–E, the expression level of YBX1 significantly increased after transfection with YBX1. Next, we examined the inhibitory effect of YBX1 on cardiomyocyte cell surface area (Fig. 5C). qRT-PCR and western blot assays indicated that Hypertrophy-related marker expression was inhibited after transfection with YBX1, as determined by Fig. 5D–F, indicating YBX1 plays an important role in pathological cardiac hypertrophy. In addition, we explored the effects of YBX1 on cardiac mitochondrial integrity and function. Overexpression of YBX1 promoted ATP production, restored mitochondrial function, and decreased ROS production (Fig. 5G–J). These results suggest that overexpression of YBX1 can significantly restore the pro-hypertrophic effect of Ang II on CMs.

### LncKCND1 regulates cardiac hypertrophy by targeting YBX1

**Fig. 6:**
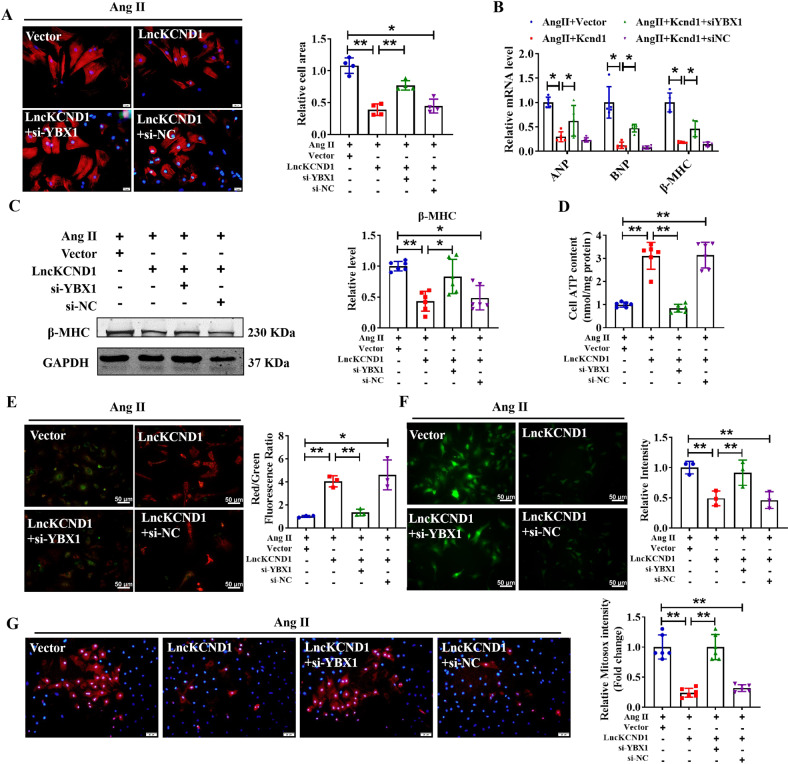
LncKCND1 regulates cardiac hypertrophy by targeting YBX1. **A** After CMs transfection with si-NC or si-YBX1 for 6 h, CMs were transfected with Vector or LncKCND1 for 6 h, then Ang II was added for 48 h. CMs were stained by α-actinin and DAPI for nuclear staining (*n* = 4, *P < 0.05, **P < 0.01; scale bars, 20 μm). **B** Relative mRNA levels of ANP, BNP and β-MHC in CMs after co-transfection (*n* = 5, *P < 0.05). **C** Representative western blot bands of the β-MHC after co-transfection of LncKCND1 and YBX1 siRNA under Ang II stimulation (*n* = 6, *P < 0.05, **P < 0.01). **D** Effect of silencing YBX1 on CMs cellular ATP content promoted by overexpression of LncKCND1 under Ang II stimulation (*n* = 6, **P < 0.01). **E** Representative images of CMs’ JC-1 staining after co-transfection (*n* = 3, *P < 0.05, **P < 0.01; scale bars, 50 μm). **F** ROS level was determined in CMs transfected with LncKCND1 and YBX1 siRNA under Ang II stimulation (*n* = 3, **P < 0.01; scale bars, 50 μm). **G** Representative images of mitochondrial ROS staining after co-transfection (*n* = 4, **P < 0.01; scale bars, 50 μm).

Recognizing that LncKCND1 can bind to YBX1 and regulate its expression in pathological cardiac hypertrophy, we hypothesized that YBX1 might be a downstream regulator of LncKCND1. Therefore, we investigated whether YBX1 could eliminate the protective effect of LncKCND1 during cardiac hypertrophy by performing rescue experiments. We first determined the silencing efficiency of YBX1 siRNA using western blot analysis. As shown in Supplementary Fig. S2F–H, the expression level of YBX1 significantly decreased after transfection with si-YBX1. We then transfected LncKCND1 and si-YBX1 into the CMs. Immunofluorescence staining of α-actinin indicated that silencing of YBX1 significantly reversed the protective effect of LncKCND1 in regulating cell size, as well as mRNA levels of ANP, BNP, and β-MHC and protein expression level of β-MHC (Fig. 6A–C). Furthermore, it can also block the recovery effect of LncKCND1 on cardiac mitochondrial function, as indicated by impaired ATP levels and increased ROS production (Fig. 6D–G). These data indicate that we have demonstrated that LncKCND1 inhibits pathological cardiac hypertrophy by targeting YBX1.

## Discussion

Cardiac hypertrophy is an adaptive response of the heart to various physiological and pathological stimuli. While physiological hypertrophy induced by exercise or pregnancy is always reversible, pathological hypertrophy observed in various cardiovascular diseases is irreversible and leads to high morbidity and mortality [18, 19]. In this study, we investigated the role of LncKCND1 in cardiac hypertrophy. We found that LncKCND1 expression levels were reduced in TAC-induced cardiac hypertrophy and hypertrophic cardiomyocytes. Knockdown of LncKCND1 promotes cardiomyocyte hypertrophy and impairs cardiac mitochondrial function. Importantly, overexpression of LncKCND1 significantly reduced the hypertrophic growth of hearts under stress stimuli, characterized by improved cardiac function, mitochondrial function, and decreased cardiomyocyte size. Moreover, we found that LncKCND1 regulated cardiac mitochondrial integrity and function by directly binding to YBX1, and protected cardiomyocytes from hypertrophy. These findings revealed a potential regulatory mechanism underlying cardiac hypertrophy.

Previous literature has reported that LncRNAs play a crucial role in cardiac hypertrophy. For example, LncRNA H19 is dysregulated in pathological cardiac hypertrophy models [20]. A recent study reported that targeting LncRNA H19 can reverse pathological cardiac hypertrophy by interacting with polycomb repressive complex 2 (PRC2) to control the nuclear factor of activated T-cells (NFAT) signaling [21]. This study identified a LncRNA AABRO7017145.1 (AAB), which was found to be upregulated in hypertrophic heart tissues and led to ferroptosis of cardiac microvascular endothelial cells by sponging and sequestering miR-30b-5p, and then resulted in an imbalance between matrix metallopeptidase 9 (MMP9) and matrix metalloproteinase 1 (MMP1) [22]. In contrast, non-coding repressor of NF18 (NRON) LncRNA was downregulated in hypertrophy-stimulated cardiomyocytes, and overexpression of NRON aggravated the hypertrophic response [23]. Interestingly, exercise-regulated cardiac LncRNA 1 (ExACT1) was found to be differentially expressed between exercised hearts and pathological hypertrophic heart tissues. While it was decreased in exercised hearts, its expression was increased in exercised hearts and cardiac hypertrophy increased in human and experimental heart failure. LncExACT1, interacted with dachsous cadherin related 2 (DCHS2), and this interaction plays an important role in the transformation between physiological and pathological growth to determine the functional outcomes [12]. Thus, our findings showed that LncKCND1 plays a crucial role in cardiac hypertrophy concurs with previous reports of pathological cardiac hypertrophy.

It is widely accepted that mitochondria provide energy to cells that require a high-energy supply, such as cardiomyocytes. Mitochondrial dysfunction induces cardiomyocyte death, pathological hypertrophy, and heart failure [24]. Thus, targeting mitochondrial function is a new strategy for treating cardiac hypertrophy. Quercetin is capable of reversing isoproterenol-induced cardiac hypertrophy by restoring cellular redox balance and protecting the mitochondria [25]. Cardiac-specific overexpression of oligomycin sensitivity conferring protein (OSCP) protects the heart from hypertrophy by improving mitochondrial function [26]. TRPC3 deficiency attenuates cardiac hypertrophy by alleviating cardiac mitochondrial dysfunction [27]. In our previous study, we found that LncRNA Plscr4 could act as a sponge for miR-214 and mediate the expression of Mfn2 to influence mitochondrial function and regulate the development of cardiac hypertrophy [15]. In this study, we demonstrated that LncKCND1 could also regulate mitochondrial function by directly binding to YBX1 in cardiac hypertrophy.

Recently, various studies have shown that YBX1 plays an important role in cardiovascular disease. YBX1 is an RNA/DNA-binding multifunctional protein that plays a critical role in regulating the physiological functions of mitochondria [28]. Consequently, it is involved in many pathological and physiological processes, such as ell apoptosis, proliferation, and translation. YBX1 interacts with overlapping sets of mitochondrial tRNAs in the cytosol under stimulation. Our result found that the overexpression of YBX1 prevented Ang II-induced mitochondrial damage in hypertrophic cardiomyocytes, which is consistent with previous reports that YBX1 depletion in cells increases mitochondrial DNA mutagenesis, leading to a decrease in the efficiency of mitochondrial repair [29, 30]. Similarly, overexpression of YBX1 prevented Ang II-induced mitochondrial damage in hypertrophic cardiomyocytes. Furthermore, overexpression of YBX1 reduced ROS levels and upregulated Ang II-induced ATP production. Ubiquitin-dependent degradation is correlated with cardiac regeneration after MI [17]. Additionally, the role of YBX1 in cardiomyocyte apoptosis remains controversial. It is said that YBX1 promotes hypoxia-induced cardiomyocyte apoptosis via SHP-1-dependent STAT3 inactivation [31], while the other study points out that YBX1 can protect cardiac myocytes against H_2_O_2_ or MI/R‑induced injury by binding to PIAS3 mRNA and resulting in the phosphorylation of STAT3 [32]. The role of YBX1 in cardiac hypertrophy has rarely been reported. In this study, we found that YBX1 expression was decreased in hypertrophic heart tissues and CMs. Furthermore, overexpression of YBX1 could markedly prevent the effects induced by Ang II.

In this study, we determined that LncKCND1 acts as an upstream regulator of YBX1 in cardiac hypertrophy. This relationship between the regulation of expression level and function of YBX1 by LncRNAs is also noticed in many other pathologies. For example, similar to LncRNA, GAS6-AS1 directly binds to YBX1 and inhibits cellular propagation, and leads to acute myeloid leukemia (AML) [33]. Similarly, LncRNA HIF1A-AS1 recruits p-YBX1 to HIF1α mRNA and consequently promotes the translation of HIF1α in pancreatic cancer [34]. Moreover, the LncRNA BASP1-AS1 regulates the transcription of Notch3 by interacting with YBX1 in melanoma [35]. Thus, from the disease point of view, it is crucial to further research the relationship between LncKCND1 and YBX1; and their role in inhibiting pathological cardiac hypertrophy in live subjects should be investigated in future studies.

**Fig. 7:**
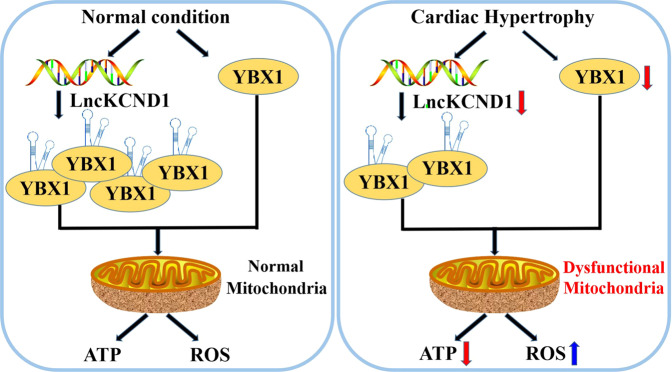
A putative model of regulation of cardiac hypertrophy by LncKCND1 targeting YBX1. During cardiac hypertrophy, the expression of LncKCND1 decreases and then leads to CMs’ mitochondrial dysfunction and aggravated cardiac remodeling by targeting YBX1. Overexpression of LncKCND1 would significantly protect CMs and hearts from hypertrophic damage, indicating a potential target for the treatment of cardiac hypertrophy.

In conclusion, our results indicated that LncKCND1 interacted with YBX1 and played an important role in cardiac hypertrophy by modulating cardiac mitochondrial function (Fig. 7). This finding encourages the possibility that LncKCND1 is a potential therapeutic target for the treatment of pathological cardiac hypertrophy.

## Supplementary information

Supplementary materials

Supplementary Figure 1

Supplementary Figure 2

Supplementary Figure 3

Supplementary Figure 4

Reproducibility checklist

## Data Availability

Data in this study are available upon reasonable request from the corresponding author at shanhl@sues.edu.cn.
