# Supplementary material for: Long non-coding RNA KCND1 protects hearts from hypertrophy by targeting YBX1
Source: Cell Death Dis. 2023 May 30;14(5):344. doi: 10.1038/s41419-023-05852-7 (PMC10229629; doi:10.1038/s41419-023-05852-7)
Supplement: Supplementary file 1 — Supplementary materials [file 41419_2023_5852_MOESM1_ESM.docx]

**Supplementary Figure S1.** (A) Cardiac function of the TAC mice was determined by two-dimensional M-mode echocardiography. (B-C) Echocardiographic assessment of left ventricle ejection fraction and fractional shortening in mice subjected to Sham or TAC surgery for 4 weeks (n = 4, ***P* < 0.01 *vs.* Sham). (D) Hypertrophy markers such as atrial natriuretic peptide (ANP), brain natriuretic peptide (BNP), and β-myosin heavy chain (β-MHC) mRNA levels in the hearts of Sham and TAC mice (n = 3, **P* < 0.05, ***P* < 0.01 *vs.* Sham). (E) Representative western blot show the expression level of β-MHC in heart from Sham and TAC mice (n = 6, ***P* < 0.01 *vs.* Sham). (F) Representative images of cardiomyocytes treated with phosphate-buffered saline or Ang II for 48 hours. Cells were identified by α-actinin staining and DAPI for nuclear staining (n = 6, ***P* < 0.01 *vs.* Control; Scale bars, 5 μm). (G) Relative mRNA levels of ANP, BNP and β-MHC in CMs treated with PBS or Ang II (n = 5, ***P* < 0.01 *vs.* Control). (H) Representative western blot bands of the β-MHC level in the CMs treated with PBS or Ang II for 48 hours (n = 6; ***P* < 0.01 *vs.* Control).

**Supplementary Figure S2.** (A) Verification of silencing efficiency of different siRNAs. CMs were transfected with si-NC or LncKCND1 siRNA for 48 hours. The most effective siRNA-3 was chosen in the following study (n=4, **P* < 0.05, ***P* < 0.01 *vs.* si-NC). (B) Overexpression of LncKCND1 was confirmed by qRT-PCR. CMs were transfected with Vector or LncKCND1 for 48 hours (n = 3, ***P* < 0.01 *vs.* Vector). (C-D) Overexpression of YBX1 was confirmed by western blot. CMs were transfected with Vector or YBX1 for 48 hours (n = 5, **P* < 0.05 *vs.* Vector). (E) Overexpression of YBX1 was confirmed by qRT-PCR. CMs were transfected with Vector or YBX1 for 48 hours (n = 3, **P* < 0.05 *vs.* Vector). (F) Silencing of YBX1 was confirmed by qRT-PCR. CMs were transfected with si-NC or YBX1 siRNA for 48 hours (n = 3, **P* < 0.05 *vs.* Vector). (G-H) Silencing of YBX1 was confirmed by western blot. CMs were transfected with si-NC or YBX1 siRNA for 48 hours (n = 6, **P* < 0.05 *vs.* Vector).

**Supplementary Figure S3.** (A) Changes of cardiomyocyte viability after 72h of AngII treatment (n = 3, **P* < 0.05, ***P* < 0.01 *vs.* 0h). (B) Changes of cardiomyocyte viability after 48h of LncKCND1 silencing (n = 6, ***P* < 0.01 *vs.* si-NC). (C) Changes of cardiomyocyte viability after 48h of LncKCND1 overexpression (n = 6, ***P* < 0.01 *vs.* Vector). (D) Changes of cardiomyocyte viability after 48h of LncKCND1 silencing (n = 6, ***P* < 0.01 *vs.* si-NC). (E) Changes of cardiomyocyte viability after 48h of YBX1 overexpression (n = 6, ***P* < 0.01 *vs.* Vector). (F) Changes of cardiomyocyte viability after transfection with LncKCND1 and YBX1 siRNA under Ang II stimulation (n = 6, ***P* < 0.01).

**Supplementary Figure S4.** (A) The expression of LncKCND1 after YBX1 overexpression (n = 5, ***P* < 0.01 *vs.* Vector). (B) The expression of LncKCND1 after YBX1 silencing (n = 5, ***P* < 0.01 *vs.* si-NC).

**Table S1 Primers used for the qRT-PCR analysis**

| **RNA name** | **Primers from 5’ to 3’** |
| --- | --- |
| LncKCND1-F | CTGCAGCGGGGACACAG |
| LncKCND1-R | CGAGGAACTGGCTGTGTGTG |
| YBX1-F | AAGGTCATCGCAACGAAGGTT |
| YBX1-R | CAAATACGTCTTCCTTGGTGTCA |
| ANP-F | ACCTGCTAGACCACCTGGAG |
| ANP-R | CCTTGGCTGTTATCTTCGGTACCGG |
| BNP-F | GAGGTCACTCCTATCCTCTGG |
| BNP-R | GCCATTTCCTCCGACTTTTCTC |
| β-MHC-F | CCGAGTCCCAGGTCAACAA |
| β-MHC-R | CTTCACGGGCACCCTTGGA |
| GAPDH-F | TCTACATGTTCCAGTATGACTC |
| GAPDH-R | ACTCCACGACATACTCAGCACC |
