# Supplementary figures and images for: Long non-coding RNA KCND1 protects hearts from hypertrophy by targeting YBX1
Source: Cell Death Dis. 2023 May 30;14(5):344. doi: 10.1038/s41419-023-05852-7 (PMC10229629; doi:10.1038/s41419-023-05852-7)

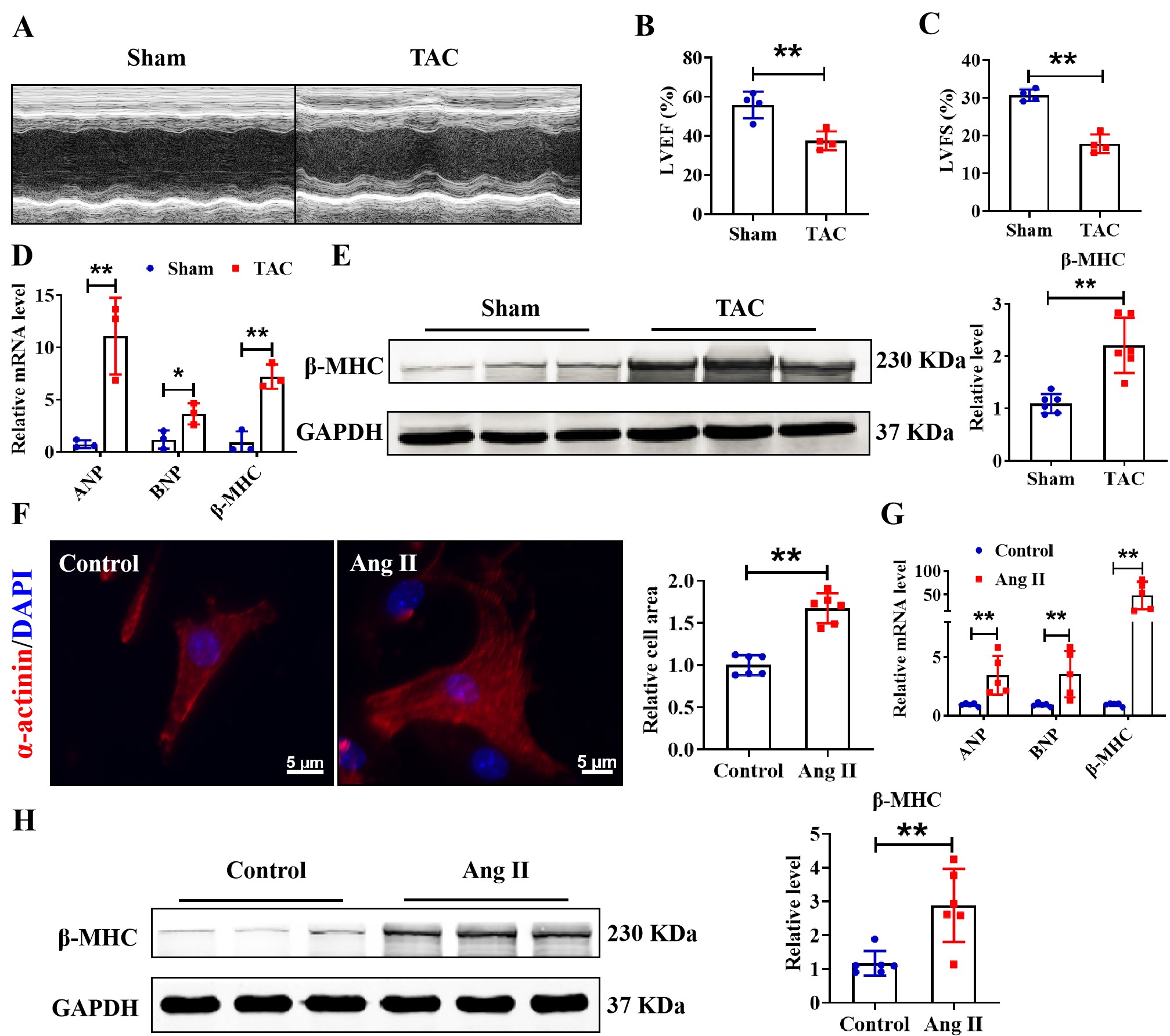

Supplement: Supplementary file 2 — Supplementary Figure 1 [file 41419_2023_5852_MOESM2_ESM.jpg]

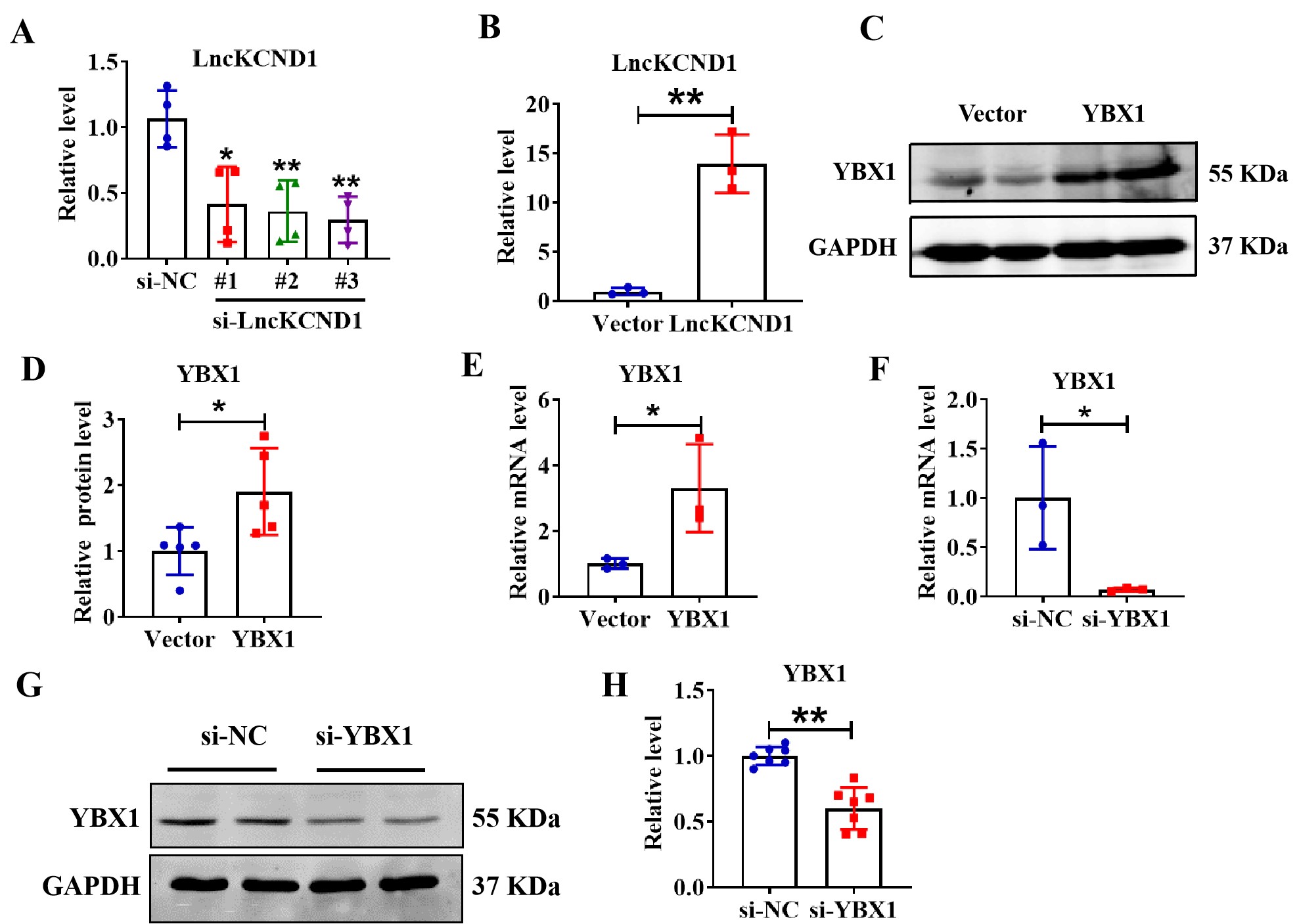

Supplement: Supplementary file 3 — Supplementary Figure 2 [file 41419_2023_5852_MOESM3_ESM.jpg]

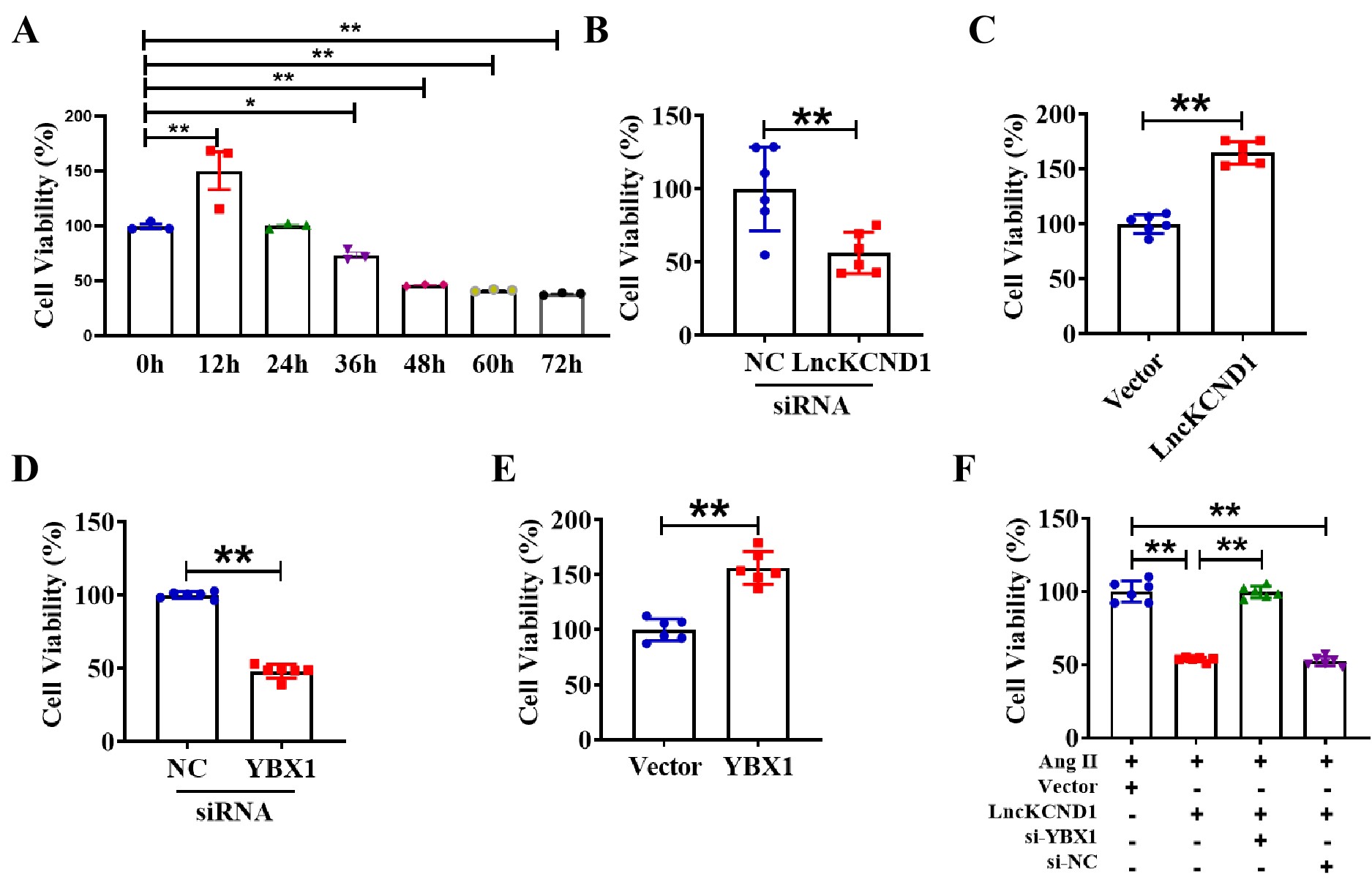

Supplement: Supplementary file 4 — Supplementary Figure 3 [file 41419_2023_5852_MOESM4_ESM.jpg]

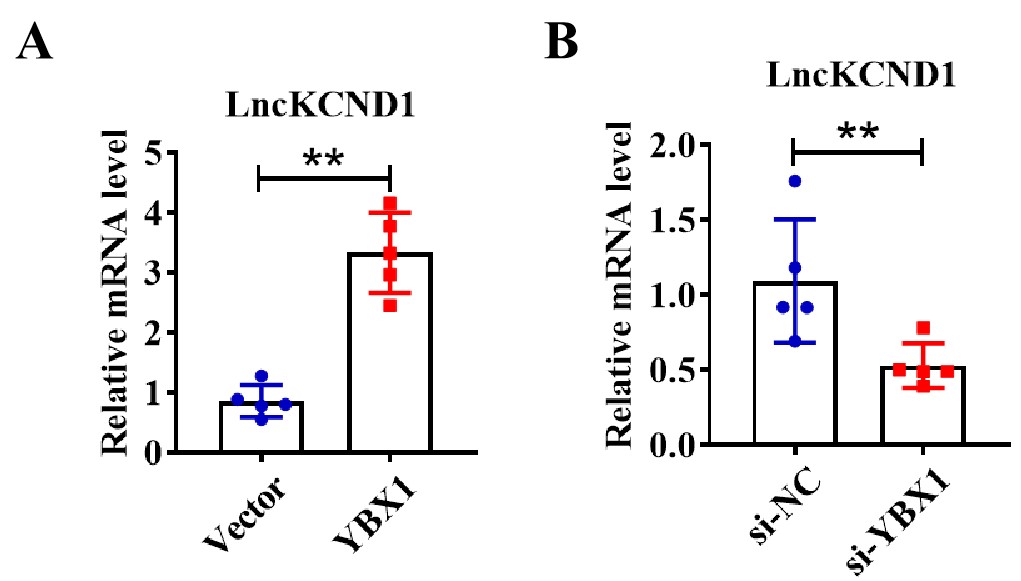

Supplement: Supplementary file 5 — Supplementary Figure 4 [file 41419_2023_5852_MOESM5_ESM.jpg]
